# GobhiSet: Dataset of raw, manually, and automatically annotated RGB images across phenology of *Brassica oleracea* var. *Botrytis*

**DOI:** 10.1016/j.dib.2024.110506

**Published:** 2024-05-15

**Authors:** Shubham Rana, Mariano Crimaldi, Domenico Barretta, Petronia Carillo, Valerio Cirillo, Albino Maggio, Fabrizio Sarghini, Salvatore Gerbino

**Affiliations:** a Department of Engineering, University of Campania “L. Vanvitelli”, Via Roma 29, Aversa, (CE) 81031, Italy; b Department of Biological and Pharmaceutical Environmental Sciences and Technologies, University of Campania “L. Vanvitelli”, Via Antonio Vivaldi, 43, 81100 Caserta, (CE), Italy; c Department of Agricultural Sciences, University of Naples “Federico II”, Via Università 100, Portici (NA) 80055, Italy

**Keywords:** *Brassica oleracea*, Manual annotation, Automatic annotation, Segment anything model, Grounding DINO

## Abstract

This research introduces an extensive dataset of unprocessed aerial RGB images and orthomosaics of Brassica oleracea crops, captured via a DJI Phantom 4. The dataset, publicly accessible, comprises 244 raw RGB images, acquired over six distinct dates in October and November of 2020 as well as 6 orthomosaics from an experimental farm located in Portici, Italy. The images, uniformly distributed across crop spaces, have undergone both manual and automatic annotations, to facilitate the detection, segmentation, and growth modelling of crops. Manual annotations were performed using bounding boxes via the Visual Geometry Group Image Annotator (VIA) and exported in the Common Objects in Context (COCO) segmentation format. The automated annotations were generated using a framework of Grounding DINO + Segment Anything Model (SAM) facilitated by YOLOv8x-seg pretrained weights obtained after training manually annotated images dated 8 October, 21 October, and 29 October 2020. The automated annotations were archived in Pascal Visual Object Classes (PASCAL VOC) format. Seven classes, designated as Row 1 through Row 7, have been identified for crop labelling. Additional attributes such as individual crop ID and the repetitiveness of individual crop specimens are delineated in the Comma Separated Values (CSV) version of the manual annotation. This dataset not only furnishes annotation information but also assists in the refinement of various machine learning models, thereby contributing significantly to the field of smart agriculture. The transparency and reproducibility of the processes are ensured by making the utilized codes accessible. This research marks a significant stride in leveraging technology for vision-based crop growth monitoring.

Specifications Table

**Table:** 

Subject	Computer Vision and Pattern Recognition, Artificial Intelligence, Agronomy and Crop Science
Specific subject area	Object detection, Object Segmentation, Automatic Annotation, Precision Agriculture, Deep Learning
Type of data	Raw images: RGB image(s) in Joint Photographic Experts Group (JPG) Dates and respective number of raw images considered for study: 8 October 2020 – 45 21 October 2020 – 35 29 October 2020 – 53 11 November 2020 – 32 18 November 2020 – 40 25 November 2020 – 39 Orthomosaics: 6 JPG files for every group of images acquired over a particular date as described above Manual Annotations: Common Objects in Context (COCO) Automated Annotations: Pascal Visual Object Classes Challenge (PASCAL VOC) Dates considered for manual annotations: 8 October 2020, 21 October 2020, 29 October 2020 and corresponding 3 orthomosaics Segmentation Masks: Portable Networks Graphic (PNG) Code: Python (.Py)
Technical Specifications of the Imaging Sensor	Model: DJI Phantom 4 Pro-Obsidian FOV: 94° 20 mm (35 mm format equivalent) f/2.8 focus at ∞ Shutter Speed: 8 - 1/8000 s Raw image dimension: 5472×3648 pixels Ground Sampling Distance: 0.12 cm/pixel at 4.3 m
Data collection	The images were acquired in field environments over six dates in October and November 2020, typically around noon. They were captured at nadir using a DJI Phantom 4 Pro-Obsidian. This was an aerial drone-based image acquisition activity following a linear grid path, with a forward and lateral overlap of 75 %. An orthomosaic image was created for each date. In total, 250 raw images were acquired, including 6 orthomosaic images. The flying altitude varied between 4.275 m and 4.749 m AGL due to wind turbulence. Manual annotations were made using VIA version 1.0.6. Automated annotations were generated using a combined framework of pretrained YOLOv8x-seg, Grounding DINO, and Segment Anything Model, to annotate crops across multi-date imagery. The results were exported in PASCAL VOC format. These annotations were grouped into 7 classes, with each class represented by a row. There are two scripts, both written in Python. The first script is designed to perform automated image annotations and evaluations using Grounding DINO and SAM. The second script is intended to extract binary masks based on automatic annotations, from the entire image and individual crop rows.
Data source location	Acquisition site: Department of Agriculture Sciences, University of Napoli Federico II, Portici, Italy Coordinates as per Real Time Kinematics’ feed: Latitude: 40; 48; 50.0137 Longitude: 14; 20; 47.7701 Data post-processing and storage location: Department of Engineering, University of Campania ‘Luigi Vanvitelli,’ Aversa, Italy Coordinates: 40.96846317808221, 14.208207168044456
Data accessibility	Repository name: Mendeley Data Data identification number: 10.17632/dcjjcwc5dh.3 Direct URL to data: https://data.mendeley.com/datasets/dcjjcwc5dh/3

## 1 Value of the Data

• This dataset is a collection of multi-date aerial imagery of the *Brassica oleracea var. Botrytis* crop [1]. The images were acquired between the first and seventh weeks after sowing the cauliflower, with the intention of observing its growth over this period. The images were annotated with two types of annotations: bounding boxes that encompass the crops along with the sub-soil area, and automated annotations that span only the canopy part of individual cauliflower specimens. This data is valuable to monitor growth across different dates.
• The dataset contains raw and annotated aerial RGB image data broadly classified into 5 subdirectories according to the nature of annotations and operations performed over cauliflower crops. The manual and automatic annotations are available in COCO segmentation format [2] as well as PASCAL VOC format [3] respectively.
• Manually annotated images dated 8 October, 21 October and 29 October 2020 were used for training YOLOv8x-seg [4] to facilitate groundedSAM (Grounding DINO + SAM) for generating canopy-bound annotation over cauliflower crops across all dates.
• The cauliflowers, which are contained in the segmentation masks of the aerial images and orthomosaics, are classified into 7 crop rows. The availability of row-wise segmentation masks facilitates both intra-date and inter-date class comparisons of cauliflower growth across these two modalities.
• Automated annotation was based on transfer learning approach [5] where the reference point of training the Grounded SAM [6] annotator was kept as the preceding date annotated imagery.

## 2 Objective

The primary motivation for compiling this dataset was to create a repository containing raw, manually, and automatically annotated pixel information about the crop of interest, *B. oleracea* var. *Botrytis*, also known as cauliflower, captured via aerial images and orthomosaics [1]. The underlying metadata can be utilized to improve growth modelling and monitoring of cauliflowers across different dates, as well as for intra-date comparison among different crop rows, making use of the two modalities: aerial images and orthomosaics. Automatic annotations play a vital role in agricultural imagery for deep learning by enabling efficient and accurate analysis at scale, contributing to the advancement of precision agriculture [7]. Annotating the exact periphery of the crop in aerial images is a function of time and cost. Bounding box-based annotation may include other objects or free space in the background, potentially reducing the accuracy of the datasets. This can be particularly problematic in agriculture, where precision is crucial for tasks such as acreage estimation, disease mapping, growth monitoring [8], or precision spraying [9]. To perform automatic crop-bound annotations, we trained a YOLOv8x-seg [4] before further training Grounding DINO [10] + SAM [11] based deep learning architecture, also known as GroundedSAM [6]. GroundedSAM uses Grounding DINO as an open-set object detector, enables the detection and segmentation of crop regions based on arbitrary text inputs. This aids in refining the bounding box annotations into annotations bound to the periphery of the crop. Based on the morphological differences observed in the segmentation masks of this multi-class annotated imagery, the growth of cauliflowers across different dates can be calculated. The same rate of growth can also be calculated using orthomosaics. In this manner, the discrepancy in growth over different dates between these two types of imagery can be modeled.

## 3 Data Description

**Fig. 1:**
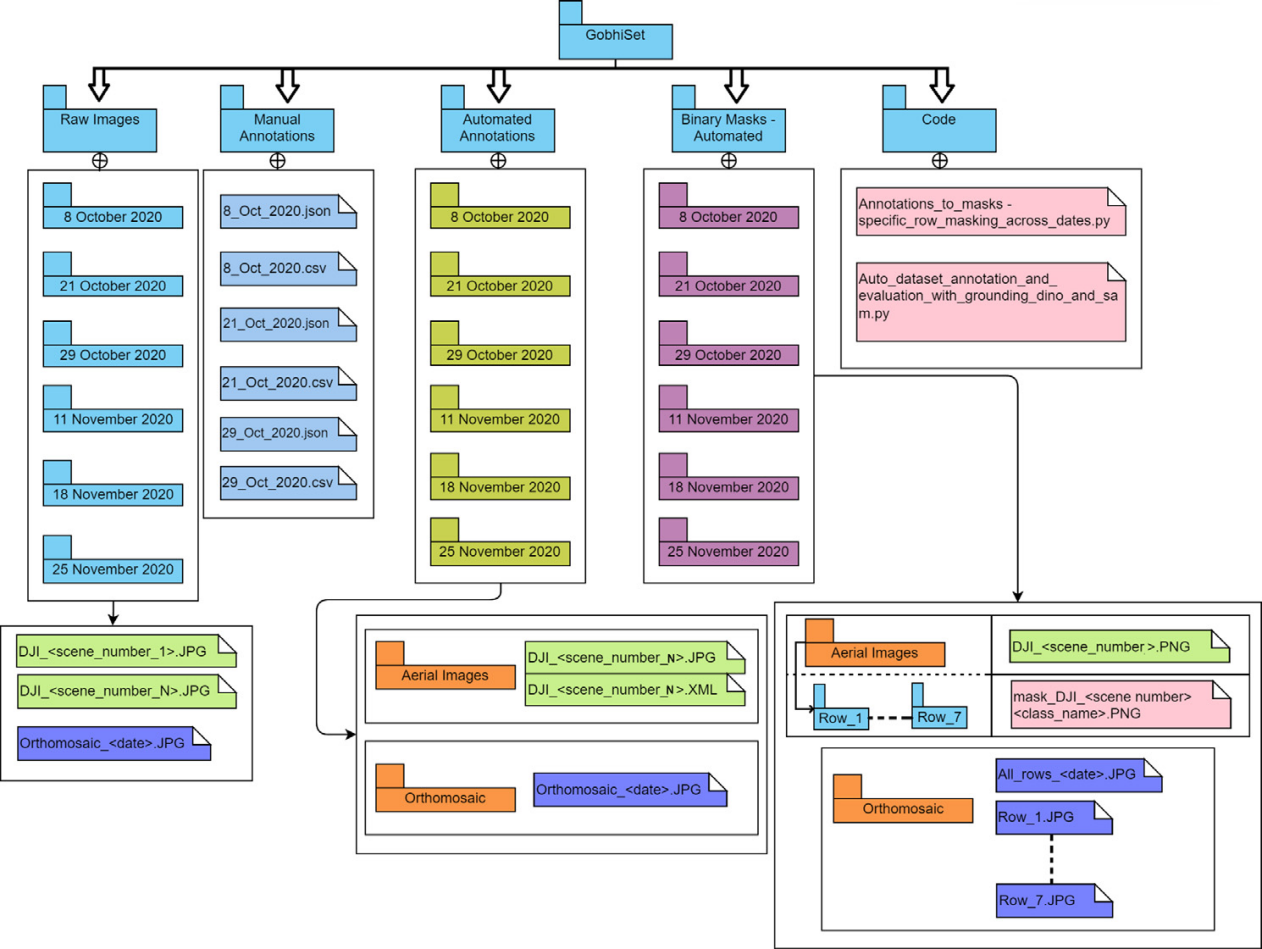
Schema of dataset.

**Fig. 2:**
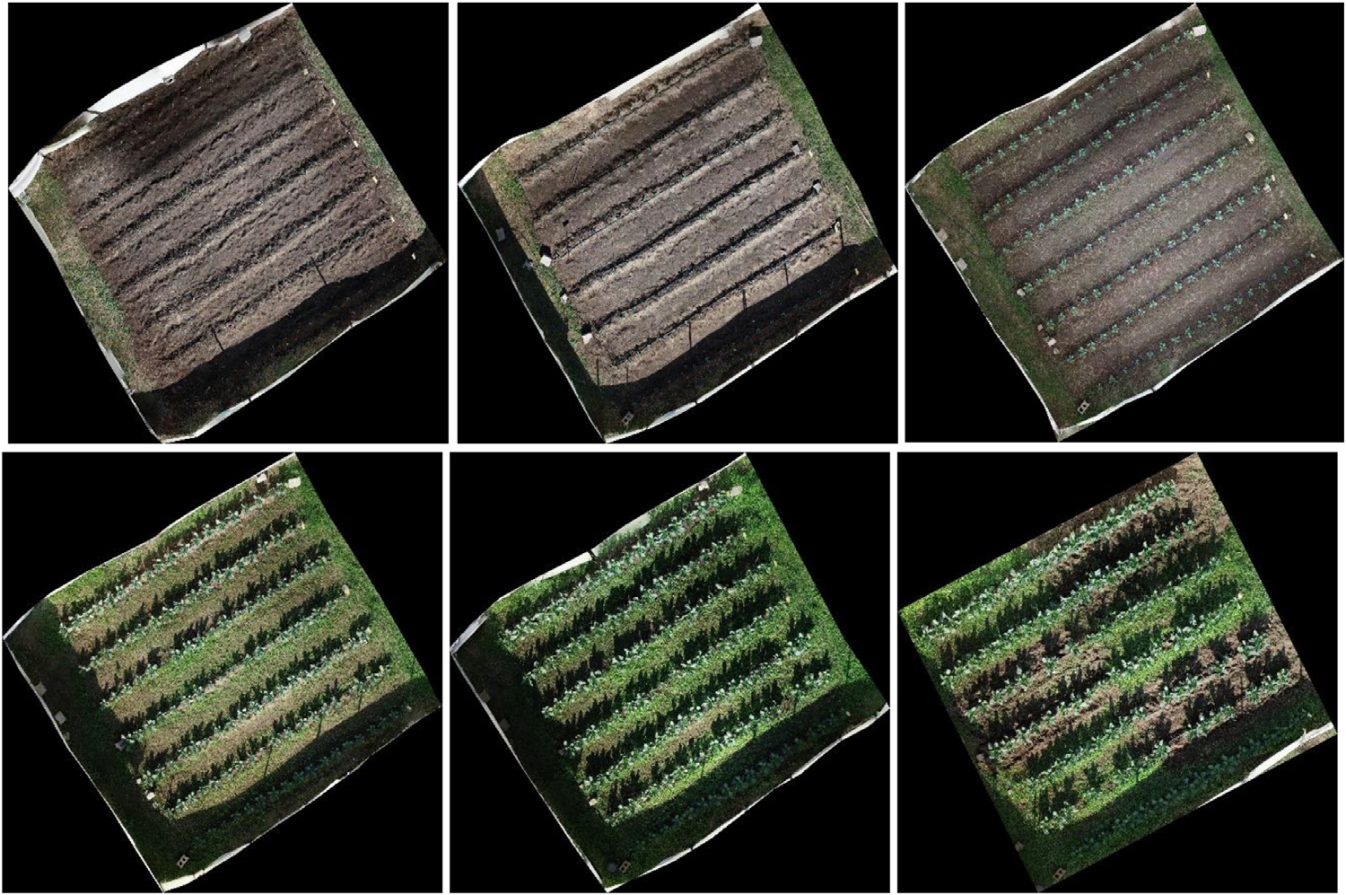
Orthomosaics of multi-date aerial RGB imagery (8 Oct, 21 Oct, 29 Oct, 11 Nov, 18 Nov, 25 Nov 2020).

**Fig. 3:**
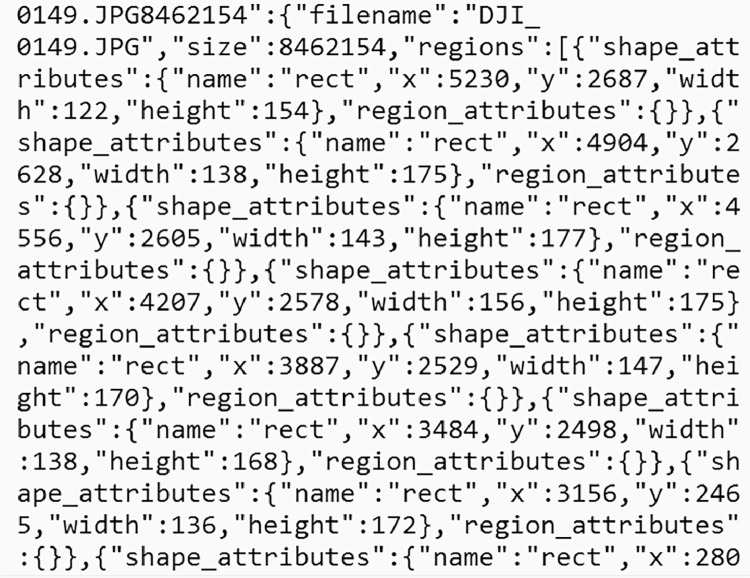
Manual annotations in COCO segmentation format.

**Fig. 4:**
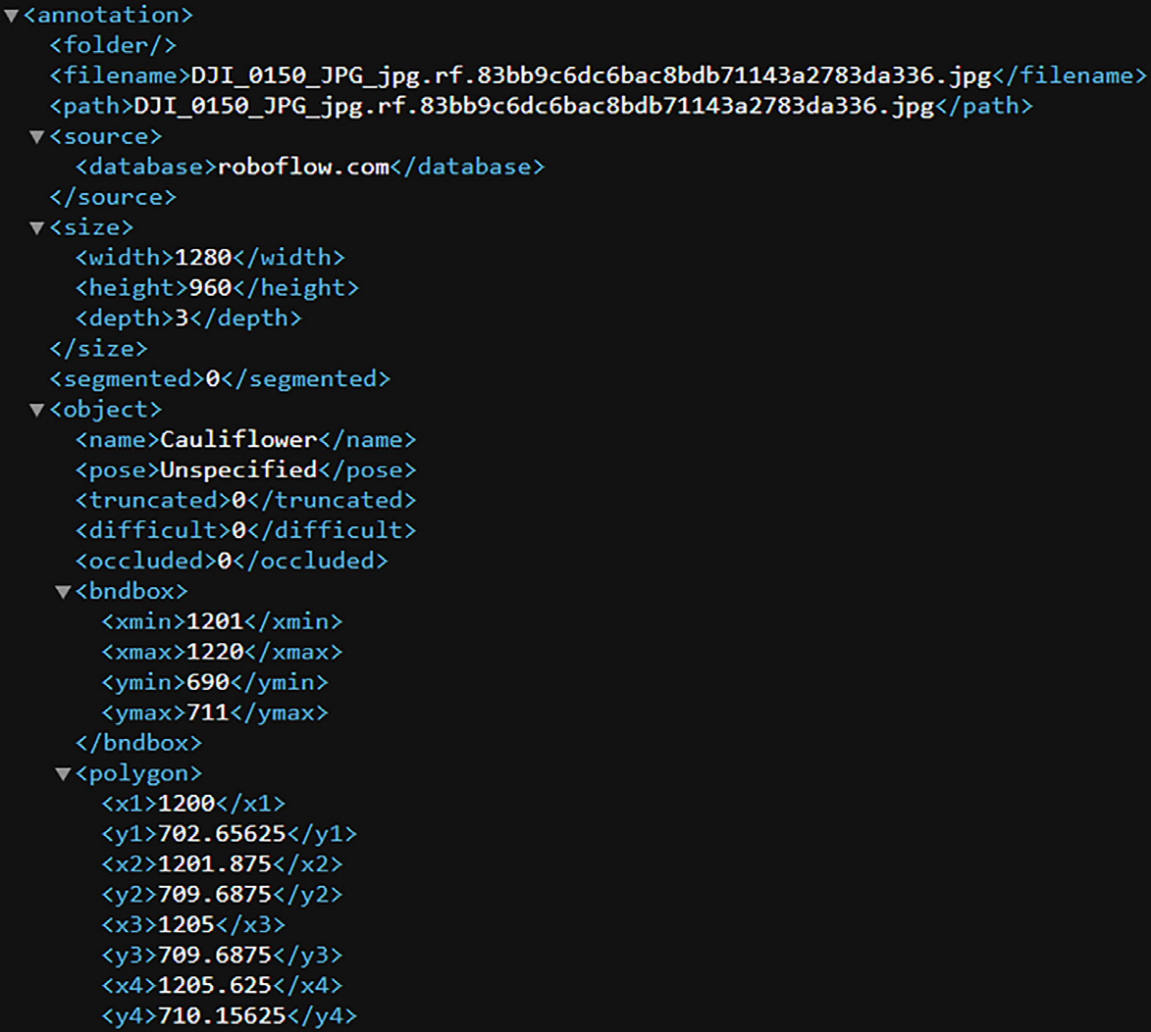
Automated annotations in Pascal VOC segmentation format.

Our dataset is publicly available in a Mendeley data library [12]. Fig. 1 shows the schema of the dataset. The cauliflower dataset ’GobhiSet’ consists of 5 products: raw RGB images, manual annotations, automated annotations, binary masks extracted from automated annotations and Python scripts to perform binary mask extraction based on specific crop rows or image and automated annotation based on bounding boxes. The **‘Raw images’** subdirectory consists of multi-date aerial RGB images of the cauliflower farm, captured in **JPG** format. The imaging was performed on 8 October, 21 October, 29 October, 11 November, 18 November, and 25 November 2020 respectively and consists of 244 images in addition to 6 multi-date orthomosaics (Fig. 2) in .JPG format. The subdirectory ‘**Manual Annotations**’ consist of six multi-date annotation files (3 COCO segmentation, 3 CSV) titled as dates on which the imaging was performed. However, the manual annotations were performed for first three dates only. These images The COCO annotation [2] file contains details about extent of bounding box used for annotations whereas the CSV annotation file contains individual crop specimen's unique identity number across all overlapping image scenes and dates. These annotations were performed in VIA annotator [13] using bounding box region shape and its nomenclature is displayed in Fig. 3. A few randomly selected sample images, their annotations and binary masks are demonstrated in Table 1. The automated annotations are saved in the PASCAL VOC format (Fig. 4) as **XML** files [3], and each annotation shares the same filename as its corresponding image, saved in **JPG** format and classified accordingly in two sub-directories: **‘Aerial Images**’ and **‘Orthomosaic’**. The segmentation masks, which are derived using automated annotations, are saved in the subdirectory **‘Binary Masks – Automated’**. These masks are in PNG format and are grouped by date. They are also classified by row numbers and stored in two sub-directories: **‘Aerial Images’** and **‘Orthomosaic’**. The former sub-directory consists of segmentation masks of all images as well as masks derived from individual row classes saved in subdirectories **‘Row_<class_name>’**. The python scripts to perform extraction of binary masks for the entire image scenes and specific rows are provided in the subdirectory **’Code’**. In a segmentation mask image, white pixels represent cauliflower, and the black pixels represent the background.

**Table 1:** Details of GobhiSet with Examples.

File Name	Subdirectories			
	Raw image	Manual annotation	Automated annotation	Binary Masks - automated
DJI_0187 (8 Oct)				
DJI_0158 (21 Oct)				
DJI_0167 (29 Oct)				
DJI_0182 (8 Oct)				
DJI_0165 (21 Oct)				
DJI_0160 (29_Oct)

## 4 Experimental Design, Materials and Methods

**Fig. 5:**
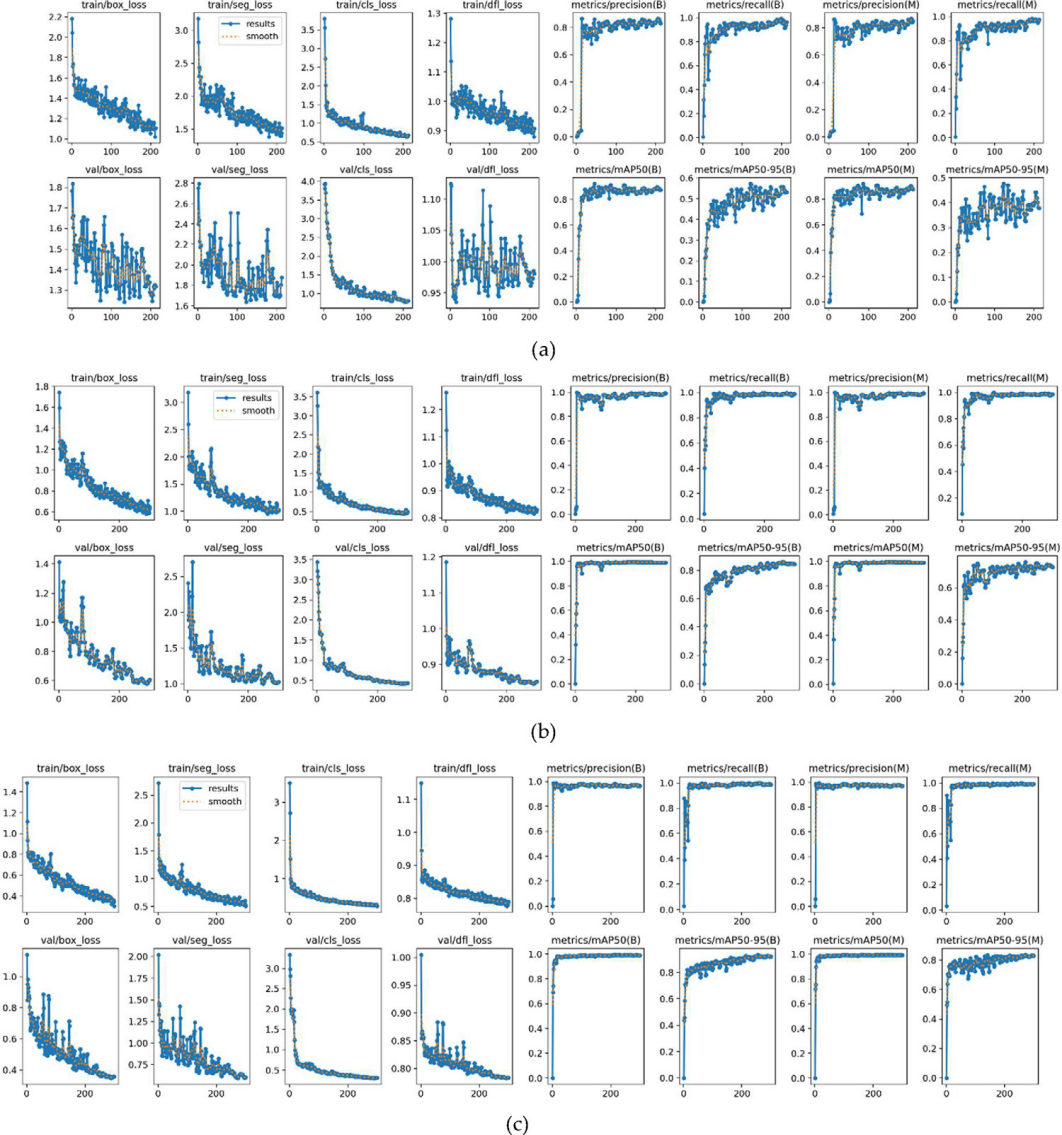
Training graphs for (a) 8 October (b) 21 October (c) 29 October 2020.

**Fig. 6:**
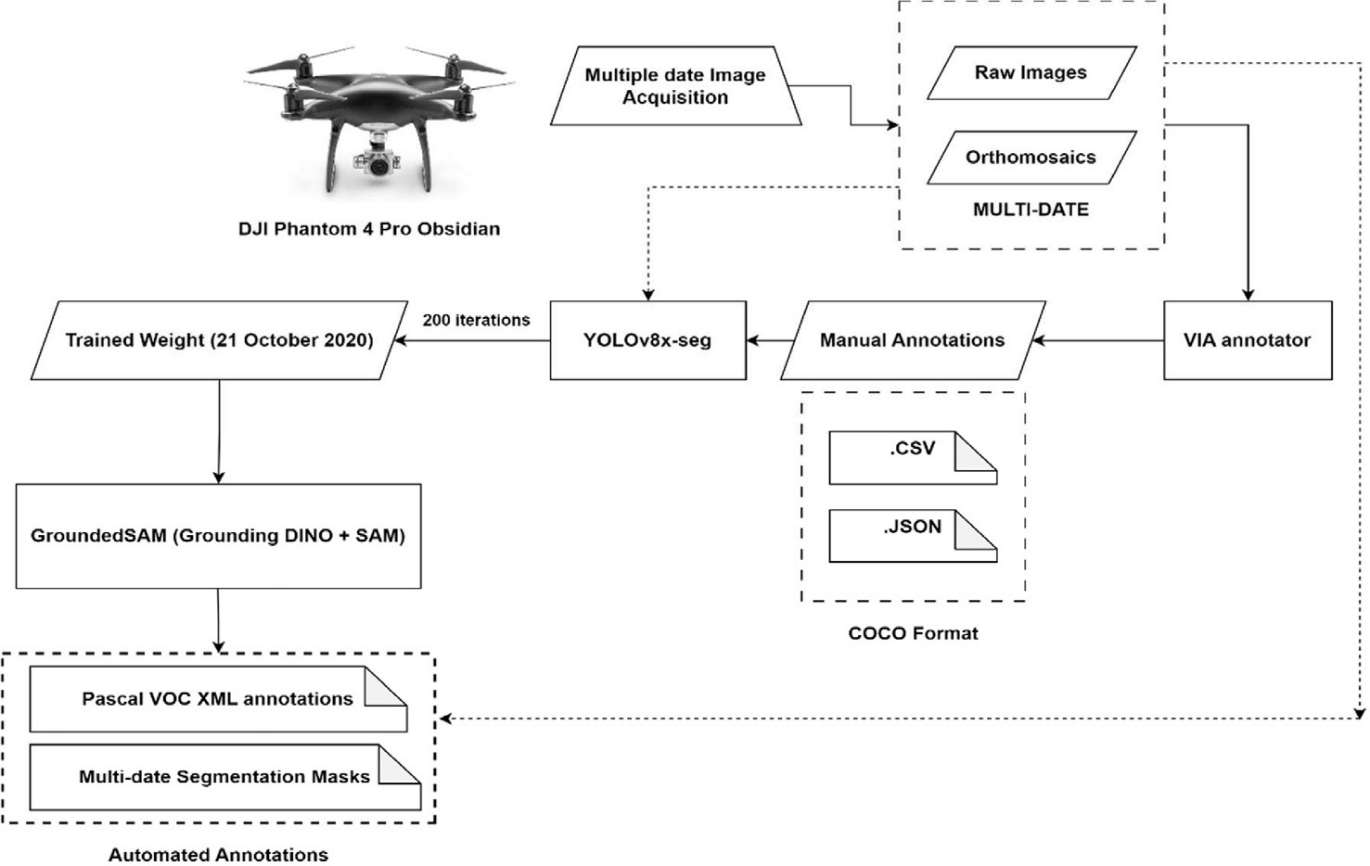
Manual and Automated annotation workflow.

The images were acquired over six different dates in the experimental farm of Department of Agriculture, University of Napoli Federico II, in Portici, Italy. This dataset covers the species of cauliflower vegetable crop, specifically *B. oleracea* var. *Botrytis*. These crops are imaged at an early stage of development, from the 1st to the 6th week after transplantation. The crop rows were neither hoed nor treated with phytosanitary products. The acquisition was performed using a DJI Phantom 4 Pro-Obsidian. The imaging was performed at nadir, with an elevation varying between 4.275 m and 4.749 m due to wind turbulence, and a forward and lateral overlap of 75 % was maintained. The orthomosaics were generated by stitching together overlapping nadir images captured by drones, making use of the image overlaps [14,15]. The lighting conditions correspond to noon time, between 11:45 and 12:30 h for every date when acquisition was performed in a field environment. The datasets dated 8 October, 21 October, and 29 October 2020 were used to independently train YOLOv8x-seg. This training generated optimal weights for tuning the hyperparameters of GroundedSAM [6]. These weights were then used to perform automated annotations across the entire batch of multi-date imagery. This process resulted in leaf-bound automated annotations for 250 multi-date RGB images and orthomosaics. The training results over 200 iterations, as depicted in Fig. 5 and detailed in Table 2, suggest that the imagery from October 21, 2020, provides the best metrics. The trained weight from this imagery is used as a benchmark in the calibration of GroundedSAM. The overall workflow is illustrated in Fig. 6.

**Table 2:** Training parameters for manually annotated imagery over 200 iterations.

Date	Training batch size	Validation batch size	Testing batch size	Mean Average Precision (mAP)	Precision	Recall
8 October 2020	30	9	6	91 %	83.8 %	91.7 %
**21 October 2020**	**23**	**6**	**6**	**99.2 %**	**99.1 %**	**98.7 %**
29 October 2020	37	11	5	99.1 %	95.9 %	99.8 %

## Limitations

A few automated annotations and corresponding segmentation masks may suffer from over-segmentation errors. For instance, boundary detection becomes difficult in the later stages of cauliflower growth, particularly in the imagery dated 11 November, 18 November, and 25 November 2020, when the leaves start overlapping each other. As a result, the crops cannot be uniquely located, and growth monitoring can be limited to a row-based approach rather than a crop-based one. Occasionally, there were infestations by predatory birds, which resulted in some crop damage in row 6 and row 7 for imagery dated 25 November 2020.

## Ethics Statement

The authors have read and follow the ethical requirements for publication in Data in Brief and confirming that the current work does not involve human subjects, animal experiments, or any data collected from social media platforms.

## CRediT authorship contribution statement

**Shubham Rana:** Conceptualization, Software, Validation, Formal analysis, Investigation, Writing – original draft, Writing – review & editing, Visualization. **Mariano Crimaldi:** Data curation, Funding acquisition, Investigation, Writing – review & editing. **Domenico Barretta:** Validation, Formal analysis. **Petronia Carillo:** Investigation, Writing – review & editing, Supervision. **Valerio Cirillo:** Supervision, Investigation. **Albino Maggio:** Resources, Project administration. **Fabrizio Sarghini:** Resources, Project administration. **Salvatore Gerbino:** Investigation, Writing – review & editing, Supervision.

## Data Availability

GobhiSet: Dataset of raw, manually and automatically annotated RGB images across phenology of Brassica oleracea var. Botrytis (Original data) (Mendeley Data)
